# Facemask performance during maxillary protraction: a finite element analysis (FEA) evaluation of load and stress distribution on Delaire facemask

**DOI:** 10.1186/s40510-018-0217-1

**Published:** 2018-07-09

**Authors:** Francesca Gazzani, Chiara Pavoni, Aldo Giancotti, Paola Cozza, Roberta Lione

**Affiliations:** 1 Department of Clinical Sciences and Translational Medicine, University of Rome ‘Tor Vergata’, Via Collazia 29, 00183 Rome, Italy; 2 Department of Dentistry, UNSBC, Tirana, Italy

**Keywords:** Delaire face mask, Maxillary protraction, Class III malocclusion, FEM analysis

## Abstract

**Background:**

To evaluate load and stress distribution on Delaire facemask (FM) during maxillary protraction in class III growing patients by means of finite element analysis (FEA). A three-dimensional geometry of a Delaire FM was reconstructed from the original CAD 3D prototype, using software package (ANSYS 5.7). FM presented forehead and chin supports and stainless steel framework characterized by two lateral vertical bars connected to a crossbar with two pawls for elastic attachment. Two traction intensities (7.8 and 9.8 N) were applied on the FM pawls along three different downward inclined directions with respect to the occlusal plane (0°, 30°, or 50°, respectively). Resulting stresses and deformations were then tested through the von Mises yield criterion in order to underline the FM wear performance.

**Results:**

The analysis showed that higher stresses and deformations are mostly related to axial forces of 9.8 N rather than 7.8 N. Stresses also progressively increased with increasing downward force inclinations (0°, 30°, and 50° with respect to the occlusal plane). The overall tensions were inferior to the limit of the elastic behavior (yield point) characterizing the material they are applied on. Thus, the FM structure absorbed the load applied with an elastic deformation of the lateral and horizontal bars.

**Conclusions:**

Resulting stresses and deformations were directly proportional to protraction load amounts and to increasing downward inclination of forces. In all tested conditions, protraction forces were not able to determine plastic deformation on FM structure compromising its performance and efficiency.

## Background

Maxillary protraction with facemask (FM) is an orthopedic approach widely used in the treatment of class III growing patients [1–8]. The FM was firstly described more than 100 years ago, but its use was lately diffused by Jean Delaire [9, 10]. Maxillary protraction therapy aims to transmit extra-oral tension forces on the circum-maxillary sutures in order to obtain a forward displacement of the maxilla stimulating bone apposition in the suture areas and resulting in an improvement of skeletal sagittal relationship [11, 12]. Although several anchorage devices have been developed [13, 14] to maximize the efficiency of the anchorage system, the Delaire FM design has never been changed over the years. Delaire FM consists of two extraoral anchorage regions, forehead and chin cups, connected to rigid and square-shaped metal framework [9, 10]. Metal component is composed of two lateral vertical bars and a crossbar with two pawls for elastic attachment [4, 9, 10]. The horizontal bar is connected to lateral vertical bars by means of two cylindrical stainless steel latches. Both the FM plastic and metallic components can be adjusted individually to adapt the FM to the size of patient’s face. Maxillary protraction usually requires 3.9 to 4.9 N of force per side with a downward inclination of 30° with respect to the occlusal plane [15–18]. Previously, Tanne et al. [19] using finite element analysis (FEA) concluded that the elastics have to be applied with a variable downward direction of about 30° to the occlusal plane in order to control the possibility of an upward displacement of the maxillary complex [6, 7, 15, 17, 19]. Although many studies [11, 20–22] have analyzed the stress and load distribution on the facial complex during maxillary protraction by means of FEA, no data are available in literature on the mechanical properties of the FM. During maxillary protraction treatment, the management of the FM components plays an important role to control the load application [17]. Differences in magnitude, direction, and duration of loads may produce several patterns of displacement and distribution of forces on maxillary complex [15–18]. Hence, the aim of the present investigation was to evaluate the dislocation and stress distribution on the FM structure by means of three-dimensional (3D) FEA in order to use the device under ideal conditions.

## Methods

In order to perform the FEA, the following parameters were defined:

- Geometrical features of the FM;
- Material properties for each element of the FM;
- Mesh (number, shape, and size of the elements used to discretize the FM)
- Constrains and loads applied on the system.

**Fig. 1:**
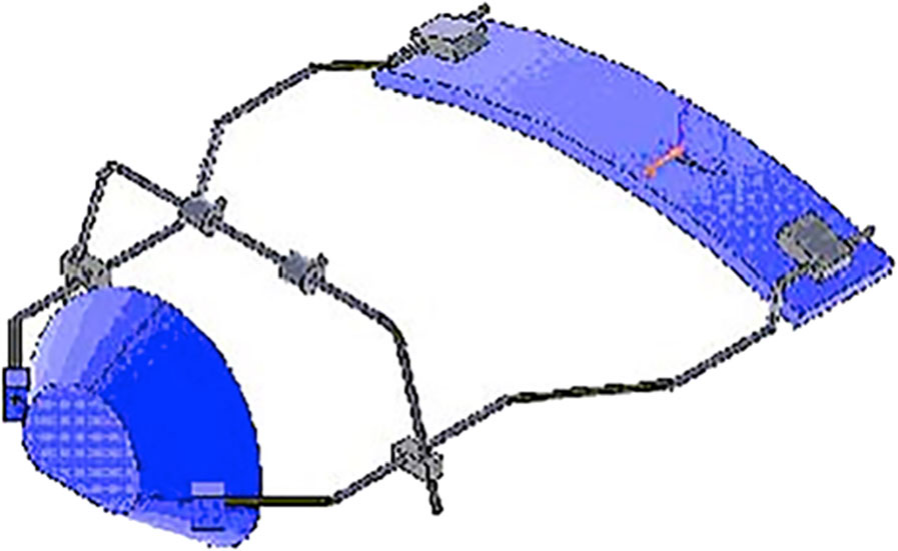
3D model of Delaire FM designed by using ANSYS 5.7

**Fig. 2:**
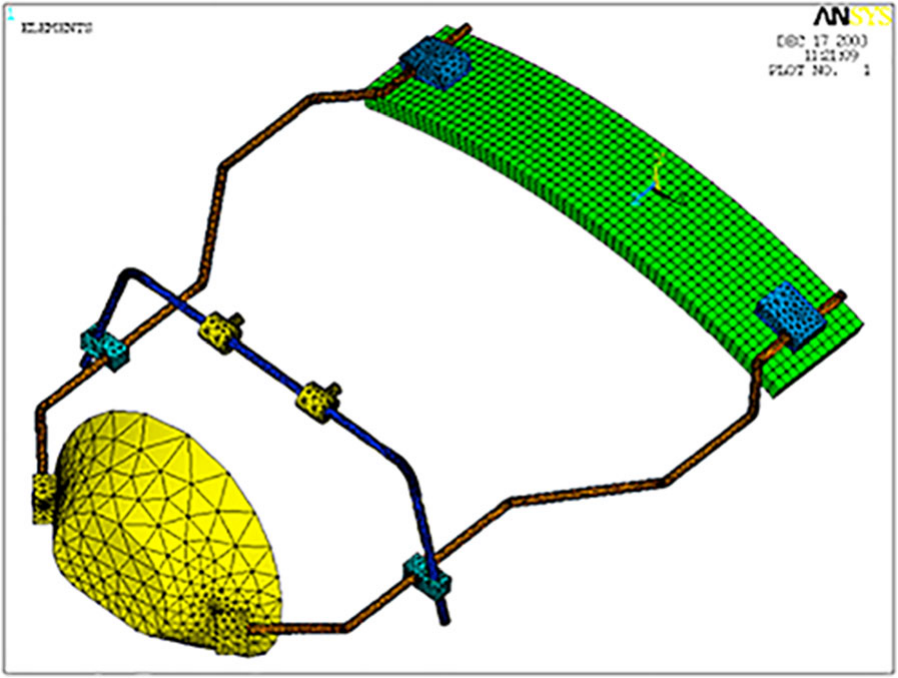
3D model meshes. Twenty-eight thousand six hundred ninety-six nodes and 40,178 elements characterized the numerical model

The ANSYS 5.7 software (Ansys Inc., Canonsburg, PA, USA) was used for the FEA. According to the input data, the software was able to solve the steady-state condition of a rigid body in the space. In particular, the system of algebraic equations was solved iteratively until the convergence of the solution is reached. As for the output data, the software evaluated the stress and strain state of the rigid body. The 3D model of a Delaire FM as originally described by Delaire (M0774-01, Leone S.p.A., Florence, Italy) was constructed and then the meshes were generated (Fig. 1). The FM structure is composed by chin and forehead supports in ABS plastic (acrylonitrile-butadiene-styrene) and stainless steel framework. ABS and stainless steel belong to different material chemistries with difference in properties. ABS is a carbon chain copolymer belonging to styrene ter-polymer chemical family. The advantage of ABS is that it combines the strength and rigidity of the acrylonitrile and styrene polymers with the toughness of the polybutadiene rubber [22]. In general, the most important mechanical properties of ABS are high tensile strength, stiffness, high impact resistance, and toughness [23, 24]. Stainless steel is a metal commonly used in orthodontics for its greater strength, higher modulus of elasticity, and good resistance to corrosion [25]. Its hardness and strength are greater when compared with ABS as shown by the increment of tensile strength and Young’s modulus (Table 2). The mechanical properties of each component were incorporated in the 3D FM structure (Table 2). The SOLID45 geometry (an element defined by eight nodes) was used for the 3D modeling of solid structures. The realized numerical model consisted of 40,178 elements (Fig. 2). Furthermore, the FEA was then conducted to evaluate different constraint and loading conditions. A FEA static simulation was performed. Thus, the behavior of the FM was studied with the application of a static load. The mesh phase and the loads were applied thanks to the interactive interface of the software. Two different traction intensities of 7.8 and 9.8 N were analyzed. The traction loads were applied with three different downward inclinations with respect to the occlusal plane (0°, 30°, or 50°) to highlight the relationship between loads, characteristics of the FM materials, constraints, and deformations. In order to underline the displacement and stress distribution on the FM structure, all the different conditions of loads and constrains were evaluated according to von Mises yield criterion. The amount of elastic energy absorption was calculated for both intensity loads (7.8 and 9.8 N) inclined of 0° to the occlusal plane in order to quantify the deformation state induced on the FM.

## Results

**Fig. 3:**
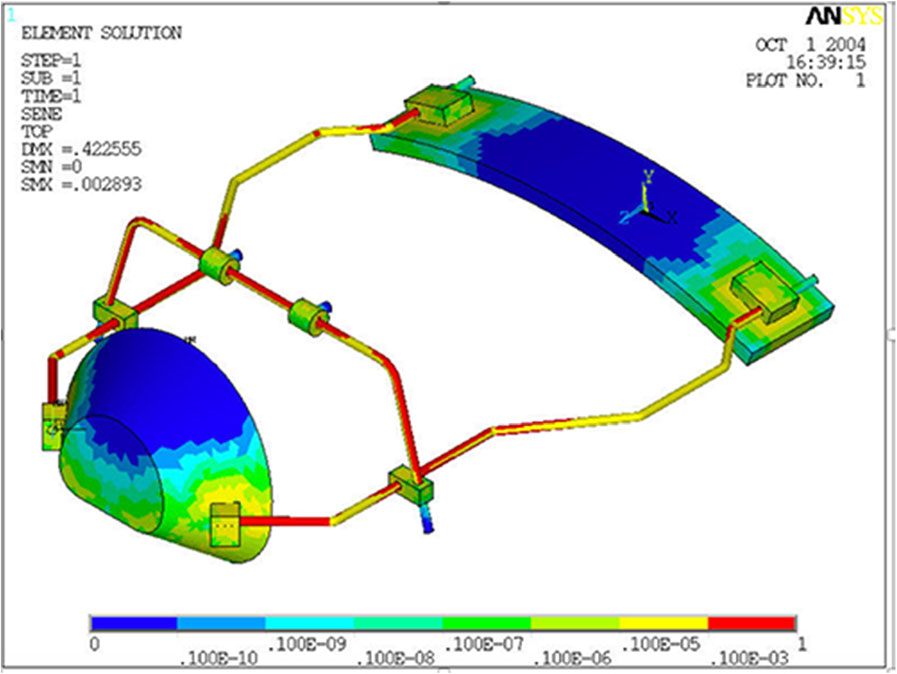
Stress distribution on the FM structure. Greater stresses were assessed on the lateral bars of the structure

**Fig. 4:**
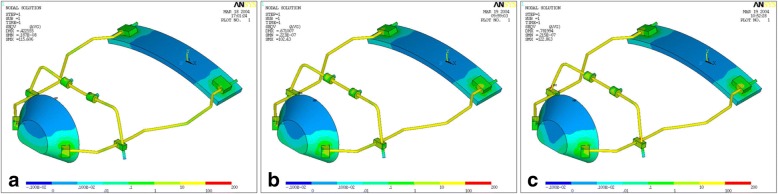
Stress distribution with traction load of 7.8 Newton applied on FM model. The force direction was progressively inclined to the occlusal plane of 0° (**a**), 30° (**b**), and 50° (**c**)

**Fig. 5:**
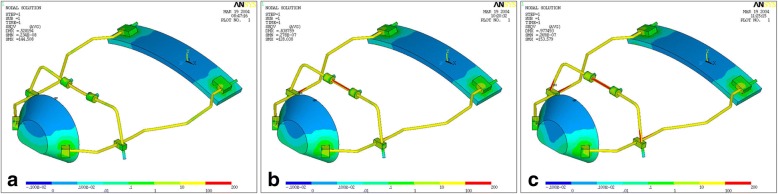
Stress distribution with traction load of 9.8 Newton applied on FM model. The force direction was progressively inclined to the occlusal plane of 0° (**a**), 30° (**b**), and 50° (**c**)

The results of the von Mises yield criterion are shown in Figs. 3, 4, and 5. The resulting tensions are reported in Newton per square millimeter (N/mm^2^). Tensile strength distribution analysis showed that greater stresses were assessed on the lateral bars of the structure (Table 1, Fig. 3). When comparing protraction intensities, the analysis showed that higher stresses and deformations are mostly related to axial forces of 9.8 N rather than 7.8 N. However, the overall tensions observed in the simulated conditions were inferior to the limit of the elastic behavior (Yield point) characterizing the material they are applied on (stainless steel limit 800 N/mm^2^; ABS limit 46 N/mm^2^) (Table 2). Stresses progressively increased with increasing downward force inclinations (respectively, 0°, 30°, and 50° to the occlusal plane). The FM structure showed an elastic behavior during its application without any implication on the own functionality.

**Table 1:** Elastic strain energy calculation after application of two traction intensities tested (7.8 and 9.8 N) with an inclination of 0° with respect to the occlusal plane

Loads (N)	Inclination	Total elastic energy [mJ]	Energy on ABS supports [mJ]	Energy on metallic lateral bars [mJ]	Energy on metallic vertical bar [mJ]
7.8	0°	1,4545	0,0090	0,8731	0,5724
9.8	0°	2,2762	0,0140	1,3643	0,8944

*N* Newton; *mJ* milliJoule, 1 mJ is equivalent to 0.001 J (Joule); *ABS* Acrilonitrile butadiene stirene

**Table 2:** Mechanical proprieties of materials

Materials	Tensile strength (N/mm^2^)	Young’s modulus (MPa)
ABS plastics	46	2100
Stainless steel	800	200,000

*ABS* Acrilonitrile butadiene stirene; *MPa* MegaPascal. 1MP a is equivalent to 1 N/mm^2^

## Discussion

Early orthopedic treatment of maxillary protraction with FM is recommended for class III growing patients [1–3, 5–7, 26]. Stresses and load distribution developed on skeletal structures during maxillary protraction were widely analyzed by means of FEA [11, 13, 27]. However, no studies exist in literature with regard to mechanical properties of FM components and their behavior under different tensile forces. Therefore, the objective of the present study was to analyze the stresses generated during maxillary protraction and their effects on FM structure. The overall results did not highlight any risks of permanent plastic deformations of the FM structures related to the variables analyzed. Regarding load conditions, the FEA confirmed that the maximum forces analyzed (9.8 N) were inferior to the tensile strength of structures’ materials (Table 2). The tensile strength is defined as the maximum tensile stress a material can endure without tearing [28]. Both plastic and metallic structures presented values of tensile strength greater than the stresses they were undergone. The maximum tensions reported on the stainless steel structure are 200 N/mm^2^ while its tensile strength is equal to 800 N/mm^2^. Similarly, the maximum tensions reported on the chin cup and on the forehead support were 10 N/mm^2^ while the tensile strength of the ABS plastic is equal to 46 N/mm^2^. The maximum stresses were observed on the connection between the horizontal and lateral vertical bars in correspondence of two cylindrical stainless steel latches. Although high stresses transmitted on the structure, the high value of the yield strength typical of stainless steel granted the absence of plastic deformation. The yield strength is defined as the amount of stress (yield point) that a material can undergo before moving from elastic deformation into plastic and permanent deformation [28]. The yield point represents the upper limit to forces that can be applied without permanent deformation. In the present investigations, the yield point of the mechanical components was always under the limit. Highest forces (9.8 N) determined greater stresses and more elastic deformations on the FM structure when compared with lower 7.8 N. However, no non-reversible plastic deformations and consequently any effects on the device efficiency were observed. A greater amount of stresses and tension loads was observed when the downward force direction was progressively inclined with respect to the occlusal plane of 0°, 30°, and 50°. The 50° inclined plane was mostly related to a plastic deformation of the structure with a more dispersion of the force applied and a fewer transmission of the forces on the maxillary complex. Thus, the clinical application of 3.9 or 4.9 N of forces per side with downward direction of about 30° to occlusal plane [29] did not determine any damaging stresses on the FM structure. Both the intensity of forces and the inclination were correlated to an increment of the stresses generated on FM structure during maxillary protraction treatment without any possibility of plastic deformation. The results obtained confirmed that the control of magnitude, direction, and duration of force is able to grant high FM performance and treatment predictability, since plastic deformation significantly influences mechanical efficiency and material performance. Thus, the clinical control of the orthopedic force and the mechanical properties of FM components preserve the structure from any risks of damage allowing to use the FM under ideal conditions.

### Clinical significance

A careful facemask management during orthopedic treatment plays an important role in granting its best performance and condition of use. Both the ABS chin and frontal supports need to be individually adjusted to fit the patient’s face maximizing the contact surface with the skin for a homogeneous distribution of the loads applied. In terms of magnitude, most studies reported that heavy forces ranging from 7.8 to 9.8 N are important to induce a more efficient maxillary growth and anterior displacement [14–18]. According to the existing literature [15–18, 29], our results showed that the most favorable force vector direction is represented by an inclination of 30° to the occlusal plane. It allows a counterclockwise rotation of the maxillary complex reducing stresses and tensile forces on the facemask device. This parameter is strictly related and conditioned to the position of horizontal bar. Therefore, it is necessary to modify the vertical position of the horizontal bar to calibrate the force vector direction setting the inclination of the elastics to 30° in downward direction. Finally, the Delaire FM did not show permanent shape changes and plastic deformations of the ABS and metallic components when undergone heavy forces preventing any risk of loads dispersion.

## Conclusions

Amount of stresses and deformations are related to load intensity and inclination of force direction. The clinical application of 3.9 or 4.9 N of forces per side with downward direction of about 30° to the occlusal plane did not determine any plastic deformation on FM structure.

## Abbreviations

ABS: Acrylonitrile-butadiene-styrene
FEA: Finite Element Analysis
FM: Face Mask
N: Newton
